# Platelet accumulation in an endothelium-coated elastic vein valve model of deep vein thrombosis is mediated by GPIb*α*—VWF interaction

**DOI:** 10.3389/fcvm.2023.1167884

**Published:** 2023-04-27

**Authors:** Hosam Alden Baksamawi, Alessio Alexiadis, Daniele Vigolo, Alexander Brill

**Affiliations:** ^1^ School of Chemical Engineering, University of Birmingham, Birmingham, United Kingdom; ^2^ School of Biomedical Engineering, The University of Sydney, Sydney, NSW, Australia; ^3^ The University of Sydney Nano Institute, The University of Sydney, Sydney, NSW, Australia; ^4^ Institute of Cardiovascular Sciences, College of Medical and Dental Sciences, University of Birmingham, Birmingham, United Kingdom

**Keywords:** DVT, microfluidics, platelets, von Willebrand factor, glycoprotein Ibα, hydrodynamics, mechanical properties

## Abstract

Deep vein thrombosis is a life-threatening disease that takes millions of people's lives worldwide. Given both technical and ethical issues of using animals in research, it is necessary to develop an appropriate *in vitro* model that would recapitulate the conditions of venous thrombus development. We present here a novel microfluidics vein-on-a-chip with moving valve leaflets to mimic the hydrodynamics in a vein, and Human Umbilical Vein Endothelial Cell (HUVEC) monolayer. A pulsatile flow pattern, typical for veins, was used in the experiments. Unstimulated human platelets, reconstituted with the whole blood, accumulated at the luminal side of the leaflet tips proportionally to the leaflet flexibility. Platelet activation by thrombin induced robust platelet accrual at the leaflet tips. Inhibition of glycoprotein (GP) IIb-IIIa did not decrease but, paradoxically, slightly increased platelet accumulation. In contrast, blockade of the interaction between platelet GPIb*α* and A1 domain of von Willebrand factor completely abolished platelet deposition. Stimulation of the endothelium with histamine, a known secretagogue of Weibel-Palade bodies, promoted platelet accrual at the basal side of the leaflets, where human thrombi are usually observed. Thus, platelet deposition depends on the leaflet flexibility, and accumulation of activated platelets at the valve leaflets is mediated by GPIb*α*-VWF interaction.

## 1. Introduction

Venous thromboembolism (VTE) is a life-threatening condition linked to cardio-vascular diseases (1, 2). VTE encompasses deep vein thrombosis (DVT), which is the formation of a thrombus in the deep veins, usually in the legs, and its most dangerous complication, pulmonary embolism (PE), which develops when the thrombus or part of it gets detached and travels to the lungs, where it occludes pulmonary circulation leading to respiratory insufficiency and even death (3–5).

DVT develops in the special milieu of venous flow, characterized by low shear and specific pulsatile flow patterns created by the muscle pump (6). Thrombi develop in the space behind venous valve leaflets, where blood can remain for significant periods, and its flow forms two unique oppositely directed vortices (7). Excessive stagnancy of blood flow is considered one of the primary and main triggers of DVT and can result from prolonged immobilization after major surgery, limb paralysis (8); or long-haul flights (9). Recently reported evidence implies that flow reduction induces a sequence of events resembling local inflammation, with the accumulation of immune cells and platelets being an essential process preceding thrombus formation. This is further confirmed by the reduced early cell recruitment in experimental animals with mutations protecting from DVT (10–12). For example, both the recruitment of cells and thrombosis are dramatically reduced and completely absent, respectively, in mice deficient for von Willebrand factor (VWF), a large pro-adhesive protein stored in platelets and Weibel-Palade bodies of the endothelium (10). This suggests the critical requirement of this protein for DVT initiation. Inhibition of platelets by low doses of aspirin successfully protects against both DVT and PE, which implies the involvement of this cell type in the pathogenesis of the disease (13).

Despite extensive research in the field, which especially intensified following the US Surgeon General's Call to Action to Prevent Deep Vein Thrombosis and Pulmonary Embolism (14), the pathogenesis of DVT remains incompletely understood. One of the reasons for this is the lack of a reliable high throughput DVT model to explore mechanisms of its initiation and propagation as well as to test the efficacy of new anti-thrombotic drugs. The existing animal models (15, 16), in which DVT is usually induced by artificial blood flow reduction in a large vein, despite their exceptional importance, have certain limitations, such as ethical issues, differences between human and rodent genomes, horizontal rather than an upright posture, and the high cost of animal purchase and maintenance. Thus, despite a number of advantages of *in vivo* models, such as the presence of multiple unrecognizable factors affecting the outcome, the possibility to work with genetically modified animals, and explore not only acute but also chronic thrombosis, the development of an *in vitro* approach that would recapitulate factors promoting DVT in all their complexity, is highly relevant and important.

Most of currently used *in vitro* models of thrombosis are primarily based on a parallel plate flow chamber, used to study cells adhesion and deposition on a surface under shear conditions (17–21). However, these models do not recapitulate the specificity of flow geometry in veins, which is defined mainly by flexible valves preventing backflow of the blood (22). Recently developed microfluidic technology has an advantage in terms of the low blood volume and number of cells needed for experiments (22, 23).

Vessel-on-a-chip models mimicking the hemodynamic microenvironment of human blood vessels is cutting-edge technology in the field of thrombosis research (24–26). The thrombosis-on-a-chip approach, recapitulating the venous environment such as specific flow patterns and endothelium, is a promising method to address mechanisms of venous thrombosis (27). In a recently published elegant study (28), authors demonstrated the initial formation of fibrin gel, followed by accumulation and activation of procoagulant platelets and thrombus growth in a microfluidics device, which combines biological (blood, tissue factor) and hemodynamic factors (valve leaflets steadily fixed at different angles) inducing thrombosis in a vein. We have recently reported a new microfluidics model with mobile valves and a pulsatile flow pattern typical for veins (29) and was validated by our in silico model (30). In the present study, we have developed a method to grow human endothelial cells on the entire surface of the channel including valve leaflets (Cellular Elastic Vein Valve model, CEVV chip). We report that (1) platelet accrual directly correlates with leaflet flexibility, and (2) platelet or endothelial activation strongly promotes platelet accumulation on the valve leaflets through glycoprotein (GP)Ib*α*—VWF interaction.

## 2. Methods

### 2.1. Master mould fabrication

The designs, along with the geometries of the microfluidic channels and the valve leaflets, have been created in AutoCAD software (Autodesk Inc, US). The design was modified to mimic the geometry of the human femoral vein and valve leaflets. The created design of microfluidic channels and valves was printed on top of a special transparency photomask (Microlithography Services Ltd., UK). The obtained photo mask is needed for the photolithography preparation of the SU8 master mould on a silicon wafer. Briefly, SU8-2075 photoresist (MicroChem, Westborough, U.S.A.) was spin-coated onto a silicon wafer (Si-Mat, Germany), and it was then exposed to ultraviolet light using a mask aligner (Canon PLA-501FA Mask Aligner) through a photo mask. The uncured photoresist was then washed away to obtain a negative master mould, with a thickness of ≈120 µm. Then, conventional photolithography techniques were used to fabricate microfluidic devices out of polydimethylsiloxane (PDMS) (Sylgard® 184 Silicone Elastomer Kit, Dow Corning, UK).

### 2.2. Preparation and modification of PDMS and PEGDA surfaces

PDMS substrates (10:1 w/w ratio with curing agent) (SYLGARD, Dow Corning, U.S.A.) were mixed well and poured over the silicon wafer master mould to create the microfluidic channels. The desired dimensions were as following: width, W, 300 μm, height, H, 120 μm and length, L, 2 cm. The PDMS was degassed under a vacuum before curing in an oven at 70°C for 2 h. Once the PDMS was cured, it was removed from the mould and used to fabricate the microfluidic device. Inlet and outlet were punched by a biopsy puncher (1.5 mm, Miltex by Kai) to create the top layer of the microfluidic device. For the bottom layer, we used a glass slide spin-coated with ≈250 μm of PDMS (30 s at 1,500 rpm) and left it to cure in an oven at 70°C for 90 min. To create the device, both layers were treated with a corona discharge for 1–1.5 min at a power of 30 Watts (PZ2 Handheld Device, Relyon Plasma GmbH, Germany) and bonded together. Then the device was placed on a hot plate for 10 min to induce irreversible bonding, followed by immersing the PDMS channel in a solution of 10% (3-Aminopropyl) triethoxysilane APTES (Sigma-Aldrich, Singapore) in Absolute ethanol and was left inside a fume cupboard at room temperature for 10 min. Next, the APTES solution was removed, and PDMS channels were washed with absolute ethanol and dried to be ready for the valve fabrication step.

For valve fabrication, the mixture of Poly(ethylene glycol) diacrylate PEGDA (Mn ≈ 700, Sigma Aldrich, UK), 0.5% Gelatine hydrolysate Enzymatic (Sigma-Aldrich, U.S.A.) in PBS and photo-initiator (2-hydroxy-2-methyl propiophenone) (Sigma Aldrich, UK) were infused into the channels. The flexible valve was created using *in situ* photo polymerization by exposing the PEGDA solution to UV light (Lumencor SOLA) for 400 ms through a photomask reproducing the valve geometry placed in the conjugated plane of an inverted microscope (Nikon Ti-U). The microfluidic devices were then cleaned and sterilized with 70% ethanol. Leaflet flexibility of the same valve was assessed visually in recorded videos by three independent observers.

### 2.3. Coating with human umbilical vein endothelial cells (HUVECs)

HUVECs were chosen for the experiments and further development of the model due to the following reasons: (1) HUVECs have endothelium structure and morphology typical for large vein, contain Weibel-Palade bodies, express a variety of endothelium-specific elements (e.g., PECAM-1, E- and P-selectins, VE-cadherin, eNOS, VWF, ICAM-1 and VCAM-1 etc.), and produce nitric oxide and prostacyclin, major endothelium-derived platelet antagonists (2) HUVECs is human endothelium from a typical large vein thus representing an adequate model to study DVT (which develops in large veins), (3) HUVECs are easily isolatable in high numbers by a non-invasive approach from a material designated as “medical waste”, and (4) A large number of published studies have been performed on HUVECs, which allows for comparison of the obtained results (31, 32). The channels were dried and immediately immersed with 2% Gelatine hydrolysate Enzymatic solution (Sigma-Aldrich, U.S.A.) in phosphate-buffered saline (PBS) and stored in the cold room at 4°C overnight. Then the devices were placed at room temperature for 1 h before the cell culture stage. Finally, the Gelatine solution was removed, and the channels were filled with the endothelial growth medium. The channel was then ready for HUVEC seeding.

HUVECs (between passages P5 and P8) were seeded at a concentration of 10⁷ ml^−1^ into the treated channels, and then incubated at 37°C and 5% CO_2_ for 4 h. The outlet and inlet were securely closed to promote cell adhesion at all the sides of the device channel and valve leaflets. To provide the HUVECs with sufficient nutrients the growth medium was perfused into the channel under a flow rate of 1 µl min^−1^. Also, this flow rate allowed for appropriate alignment of the HUVECs with the flow direction.

### 2.4. Blood preparation

All human blood experiments were performed in accordance with the principles of the Declaration of Helsinki, ethical approval granted by University of Birmingham internal ethical review (ERN_11-0175AP20) and informed consent was obtained from all donors. Blood was drawn from healthy volunteers and immediately mixed with 3.8% sodium citrate (9:1). Platelet rich plasma (PRP) was obtained by centrifugation of whole blood (200 g, 10 min). Washed platelets were obtained by centrifugation of PRP (1,000 g, 10 min) in the presence of 10 μg prostacyclin and resuspended in modified Tyrode's-HEPES Buffer (134 mM NaCl, 0.34 mM Na2HPO4, 2.9 mM KCl, 12 mM NaHCO3, 20 mM HEPES, 5 mM glucose, 1 mM MgCl2; pH 7.3). In experiments with resting (i.e., not pre-treated by any agonist or inhibitor) platelets, they were labelled by Calcein Red-Orange, AM, at 1:100 ratio, at room temperature for 20 min, and reconstituted with the whole blood. For challenged platelets experiments, platelets were treated with thrombin (0.1 U/ml), eptifibatide (9 µM) or OS-1 peptide (M3456 CTERMALHNLC, Alta Bioscience, 12 µM) for 5 min, then labelled with Calcein Red-Orange and reconstituted with the whole blood. All microfluidics experiments were performed at 37°C. To mimic typical venous flow pattern, pressure controller was set to pulse whole blood (between 0 and 120 mbar with a frequency of 1 Hz) through the valves against constant backflow.

### 2.5. Immunostaining

HUVECs within the CEVV chip were fixed with 4% paraformaldehyde for 10 min and washed carefully with Dulbecco's phosphate buffered saline (DPBS). Then the HUVECs were permeabilized with 0.1% Triton X-100 (Sigma-Aldrich) in BSA/DPBS for 15 min and blocked with 1% BSA in DPBS for 1 h at room temperature. HUVECs were labeled with CD144 (VE-cadherin) Monoclonal Antibody (16B1), eBioscience™ (Invitrogen, UK) at 1:100 dilution in 0.1% BSA, and CD31 Recombinant Rabbit Monoclonal Antibody (Invitrogen, UK) at 1:100 dilution in 0.1% BSA, incubated at 4°C overnight. Then the HUVECs monolayer were labelled with Secondary Antibody, Alexa Fluor® 488 Goat anti-Mouse (Invitrogen) at a dilution of 1:200, and Secondary Antibody, Alexa Fluor® 568 Goat anti-rabbit (Invitrogen, UK) at a dilution of 1:200 for 60 min at room temperature, then the channels were washed carefully with DPBS. Finally, nuclei were stained with Hoechst 33342 at a dilution of 1:10,000 in 0.1% BSA for 10 min at room temperature and then were washed carefully with PBS. The images were captured at 20X magnification using Zeiss confocal microscope LSM780.

### 2.6. Image quantification and statistical analysis

**Figure 1:**
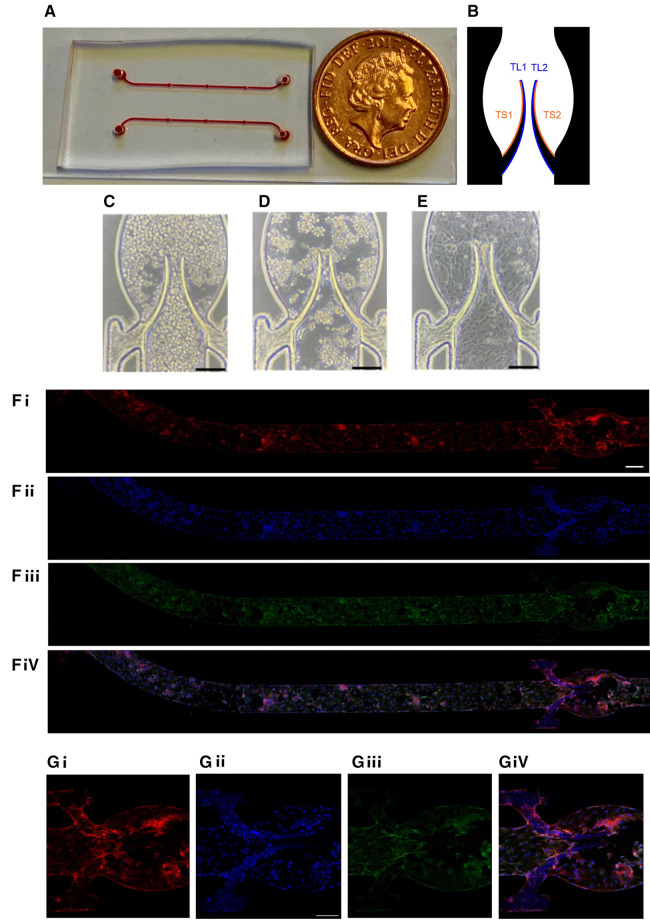
Chamber coated with endothelial monolayer. (**A**) General view of the device. (**B**) Subdivision of the valve into four areas (2 for each leaflet) for quantitation. HUVEC cells were seeded in the flow chamber (**C**) and incubated for 4 h without flow (**D**) followed by 20 h underflow (1 µl/min; E, bar 150 µm). Representative images of the entire chamber (**F**) or the valve area (**G**) stained for CD31 (**Fi,Gi**), nuclei (DAPI, **Fii,Gii**), VE-cadherin (**Fiii,Giii**) and merge (**Fiv,Giv**). Scale bar 200 and 150 µm of **F** and **G**, respectively.

Platelet accumulation was analyzed by Fiji ImageJ software (NIH, Bethesda, MD; https://imagej.net/Fiji). To measure the fluorescent area in µm², a threshold was used. For analysis purposes, each leaflet was divided into two regions, the TL area, which represents the tip, and the luminal side of the leaflet, and the TS area which represents the tip and the sinus side of the leaflet (Figure 1B). All data are presented as mean ± standard error of mean (SEM) and comparisons were performed by unpaired Student's *t*-test using GraphPad Prism (GraphPad Software Inc., USA).

## 3. Results

### 3.1. Cellular elastic vein valve model

The microfluidic device used in the experiments has some advantages vs. our previous model (29). Based on the femoral vein valve duplex images (33), the sinus area was expanded to be closer to the real shape of the human veins. Briefly, it was composed of a microchannel made of polydimethylsiloxane (PDMS) incorporating a flexible valve made of Poly(ethylene glycol) diacrylate (PEGDA) fabricated by *in situ* photo-polymerization to mimic the geometry and elasticity of venous valves. The general overview of the device is presented in Figure 1A. Platelet accumulation on basal (sinus, TS area) and luminal (TL area) surfaces of the leaflets was quantitated separately and analyzed as described in Methods (Figure 1B). The microfluidic device was coated with Human Umbilical Vein Endothelial Cells (HUVEC) for 24 h (see Methods) to reach a confluent monolayer on both the walls of the microchannel and the valve leaflets. The monolayer's continuity was confirmed by both brightfield microscopy (Figures 1C–E) and staining for endothelial markers, VE-Cadherin and PECAM-1/CD31 (Figures 1F,G). The cell monolayer was sufficiently stable to endure the shear stress used in the experiments without visible denudation.

### 3.2. Unchallenged platelets accumulate at the luminal side of leaflet tips

**Figure 2:**
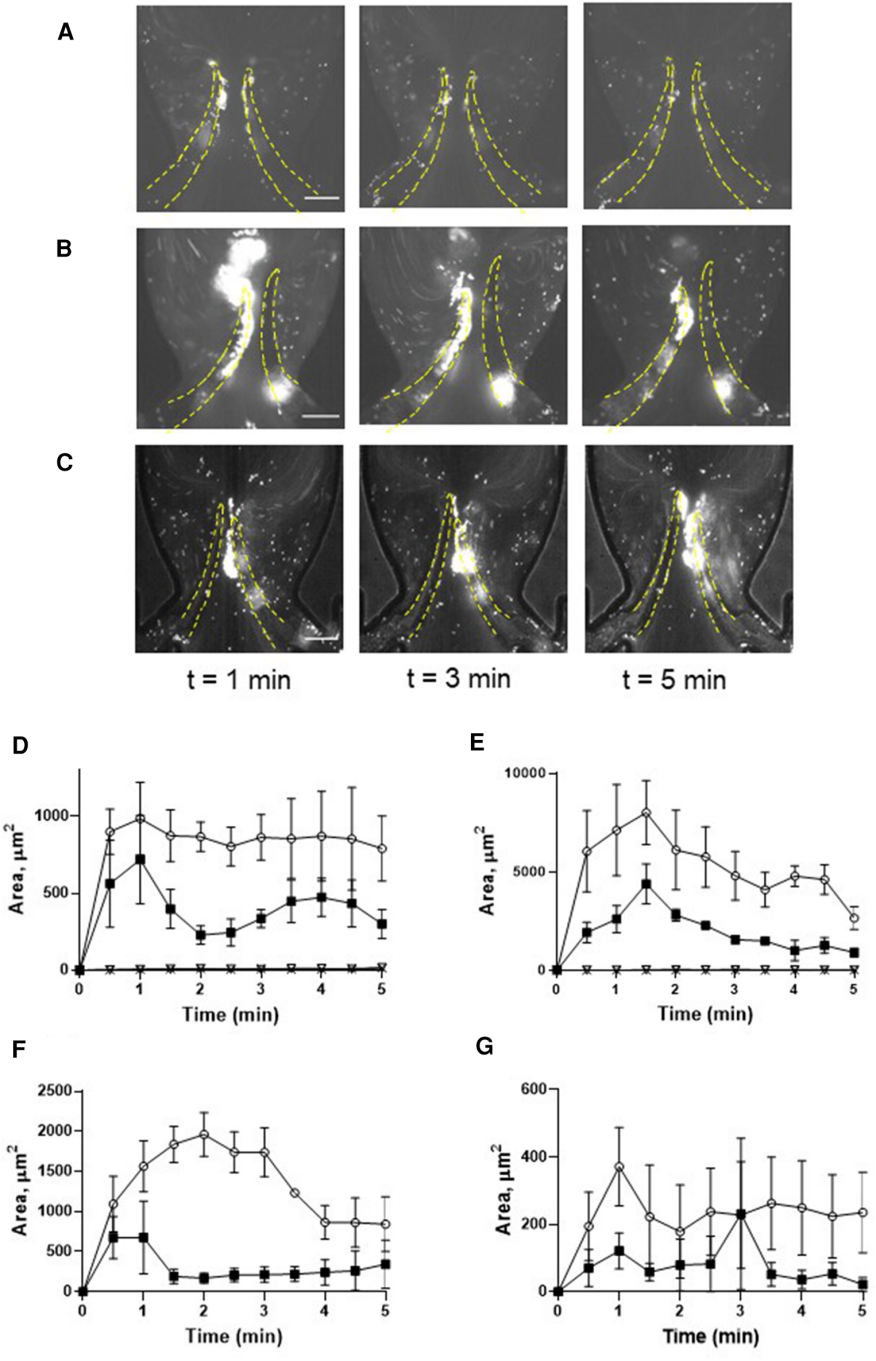
Deposition of unchallenged platelets on symmetrical and non-symmetrical valves. Blood reconstituted with fluorescently labelled platelets was perfused through either symmetrical (**A**) or non-symmetrical (**B**) valves, and resting platelets were preincubated with 9 mM eptifibatide (**C**) through a symmetrical valve for 5 min. Quantitation of resting platelet accumulation on more flexible (TL area, circles; TS area, open triangles) and less flexible/immobile (TL area, squares; TS area, Xs) leaflets of the symmetrical (**D**) or non-symmetrical (**E**) valve. (**F,G**) Quantitation of eptifibatide-treated platelet accumulation at the TL (**F**) and TS (**G**) regions. Scale bar 100 µm. Error bars represent SEM, *n* = 3.

To delineate the impact of leaflet flexibility, two types of valves were fabricated: either with both leaflets flexible (although not to an identical extent), designated as “symmetrical”, or with one leaflet flexible and another one completely rigid, defined as “non-symmetrical”. Platelet accumulation started immediately when shear was applied and reached its peak within 1 min. Perfusion of reconstituted blood through a symmetrical valve resulted in the accumulation of platelets at the luminal side of the tip, with more platelets accruing at the more flexible leaflet [area under curve (AUC) 4,126 ± 405 vs. 1,994 ± 310, *p* < 0.02; TL area, Figures 2A,D, Supplementary Movie S1]. In the non-symmetrical valve, robust platelet deposition was also observed in the TL area exclusively of the flexible leaflet (AUC 26,296 ± 2,875 vs. 9,848 ± 946, *p* < 0.006; Figures 2B,E, Supplementary Movie S2). There was no platelet accumulation in the TS area in both types of valves (Figures 2A,B,D,E; Supplementary Figures S1A,B).

Conformational change of GPIIb-IIIa (*α*IIb*β*3 integrin), resulting in acquiring an ability to bind fibrinogen, is a well-known hallmark of platelet activation by various agonists including thrombin. This facilitates the formation of a fibrinogen bridge linking adjacent platelets together, which underlies platelet aggregation (34). We therefore next assessed whether this interaction mediates platelet recruitment to the leaflets. Inhibition of *α*IIb*β*3 integrin by eptifibatide did not reduce but rather moderately enhanced platelet deposition (more flexible leaflet of symmetrical valve, AUC 4,126 ± 405 vs. 6,660 ± 513, *p* < 0.02; Figures 2C,F,G; Supplementary Movie S3; AUC of TL and TS areas is presented in Supplementary Figure S1C). In accordance with this, platelet passage through the microfluidics system did not induce platelet activation, as judged by unchanged expression of *P*-selectin, phosphatidylserine, the ability to bind PAC-1 antibody, and lack of GPIb*α* shedding (Supplementary Figure S2). Thus, similarly to the micro-channel without endothelial cells (29), platelet accumulation correlated with the degree of leaflet mobility.

### 3.3. Thrombin activation enhances platelet accumulation

**Figure 3:**
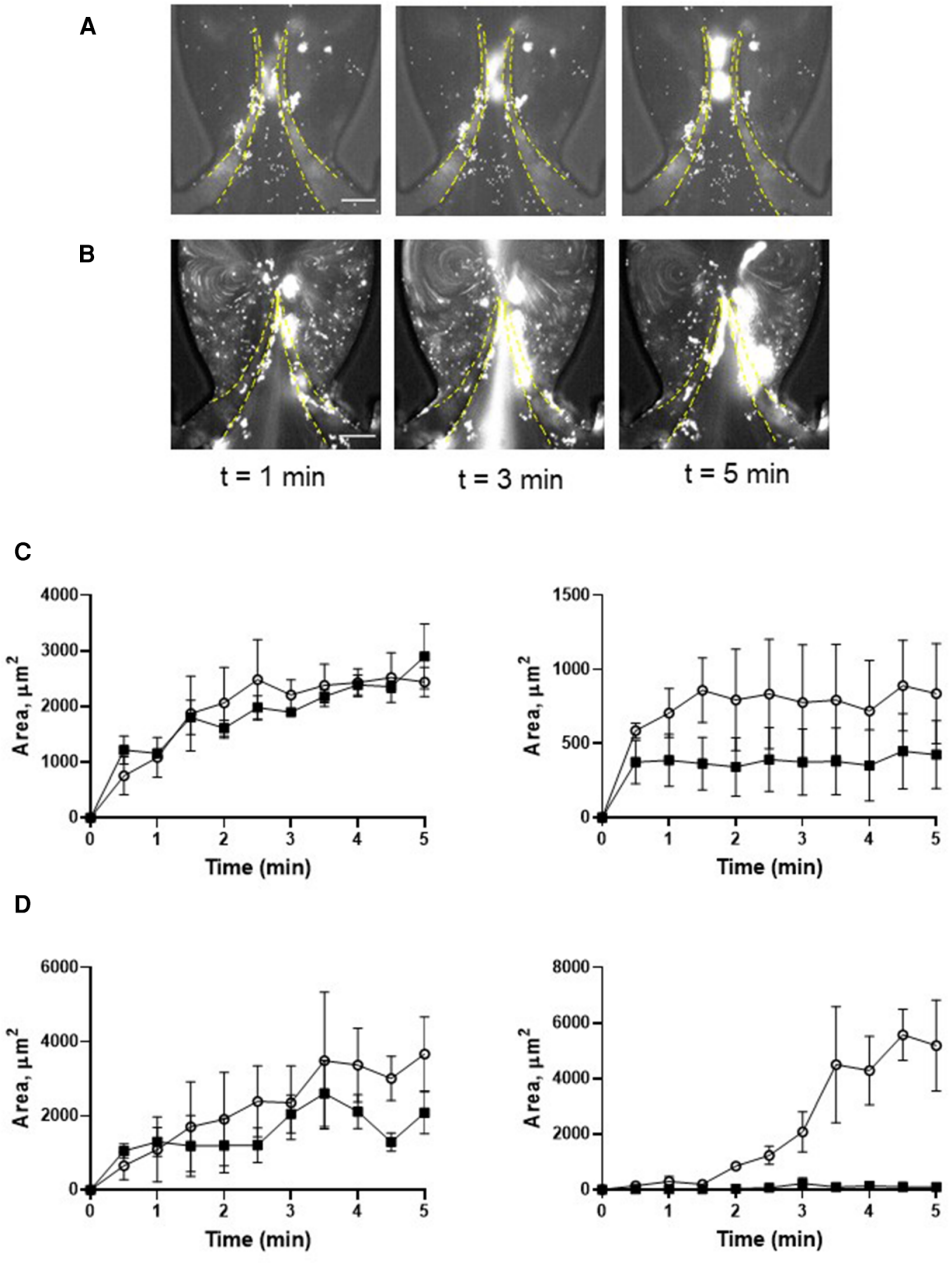
Deposition of platelets is increased by thrombin. Platelets were preincubated with 0.1 U/ml thrombin (**A,C**), and with 0.1 U/ml thrombin + 9 µM eptifibatide (**B,D**), Blood reconstituted with fluorescently labelled platelets was perfused through a symmetrical valve for 5 min. (**C,D**), left row represents TL region, and the right row represents TS region; (more flexible, circles; less flexible, squares). Scale bar 100 µm. Error bars represent SEM, *n* = 3.

Preactivation of platelets with thrombin resulted in their clumping in the flowing blood and the formation of massive “caps” on the leaflet tips (Figures 3A, C; Supplementary Movie S4; AUC of TL and TS areas is presented in Supplementary Figure S1D). The maximal TL area of the more flexible leaflet of symmetrical valve covered by platelets exceeded that for the resting platelets by ∼2 fold (AUC 4,126 ± 405 vs. 9,517 ± 896, *p* < 0.006). Thrombin-activated platelet adherence was observed also in the TS area of the leaflet (AUC 3,690 ± 578 vs. zero in resting platelets). Platelet masses remained anchored to the leaflets throughout the entire course of the experiments.

### 3.4. Accumulation of activated platelets depends on GPIb*α*—VWF A1 domain interaction

**Figure 4:**
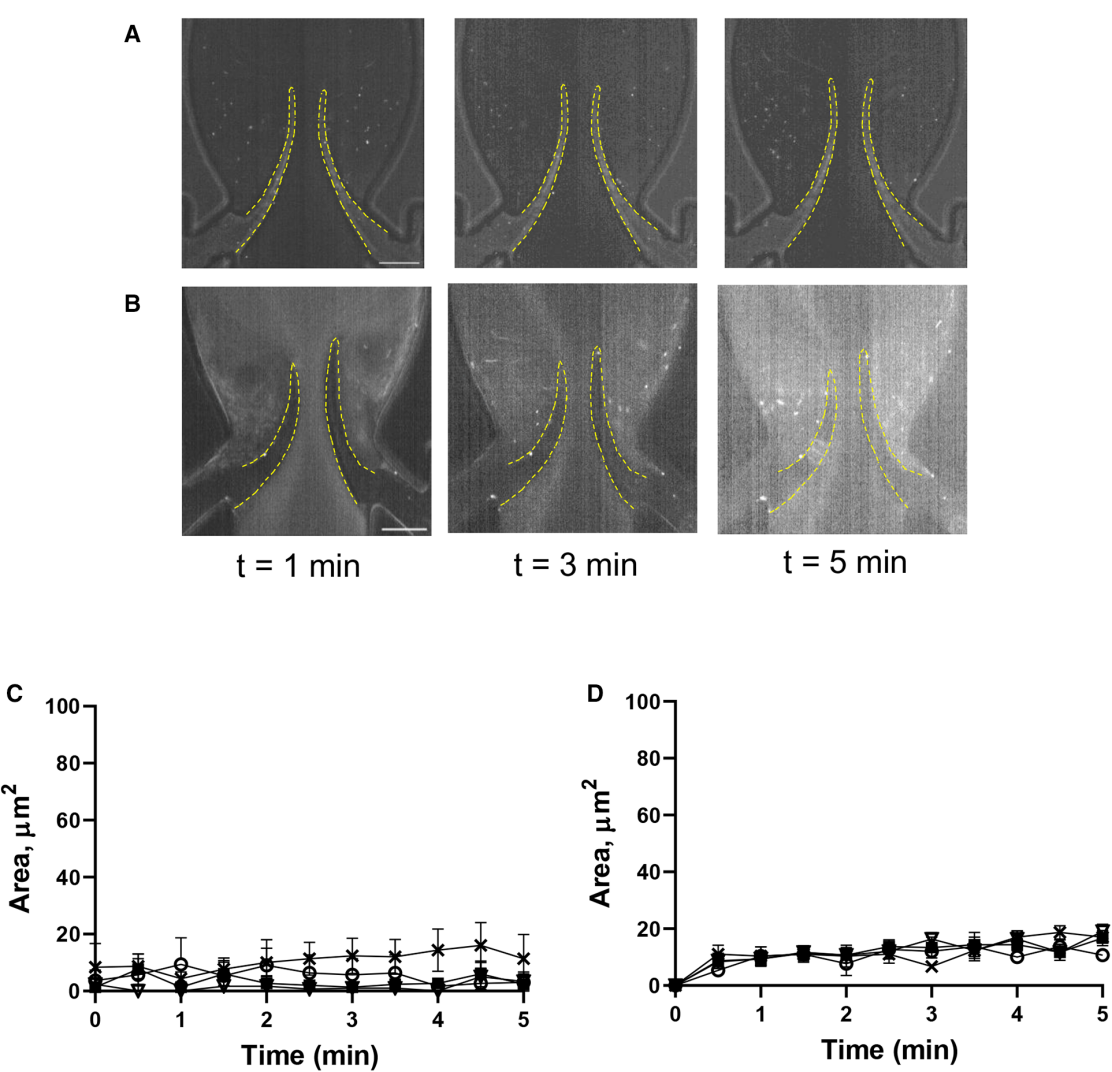
Deposition of platelets is mediated by the VWF-GPIb*α* axis. Platelets were preincubated with OS-1 peptide (M3456 CTERMALHNLC, Alta Bioscience, 12 µM) (**A**), or with 0.1 U/ml thrombin + OS-1 peptide (**B**) for 5 min, fluorescently labelled, reconstituted with whole blood, and perfused through a symmetrical valve for 5 min. (**C,D**), Quantitation of platelet accumulation on more flexible (TL area, circles; TS area, open triangles) and less flexible (TL area, squares; TS area, Xs) leaflets. Scale bar 100 µm. Error bars represent SEM, *n* = 3.

Pre-treatment of thrombin-activated platelets with eptifibatide did not reduce platelet depositions (TL area of more flexible leaflet of symmetrical valve, AUC 9,517 ± 896 vs. 10,892 ± 2,044, *p* = 0.57; Figures 3B,D; Supplementary Movie S5). Moreover, similar to resting cells, in the presence of eptifibatide, thrombin-activated platelets also accumulated behind the leaflets (TS areas; AUC of TL and TS areas is presented in Supplementary Figure S1E). This finding suggests the involvement of another mechanism of platelet accumulation under these conditions. Inhibition of the interaction of platelet GPIb*α* with VWF A1 domain with OS-1 peptide (35) of either resting or thrombin-activated platelets resulted in complete abolishment of platelet deposition on all parts of the leaflets (Figures 4A–D; Supplementary Movie S6,7; AUC of TL and TS areas is presented in Supplementary Figures S1F,G).

### 3.5. Endothelial activation promotes platelet accumulation at the space behind valve leaflets

**Figure 5:**
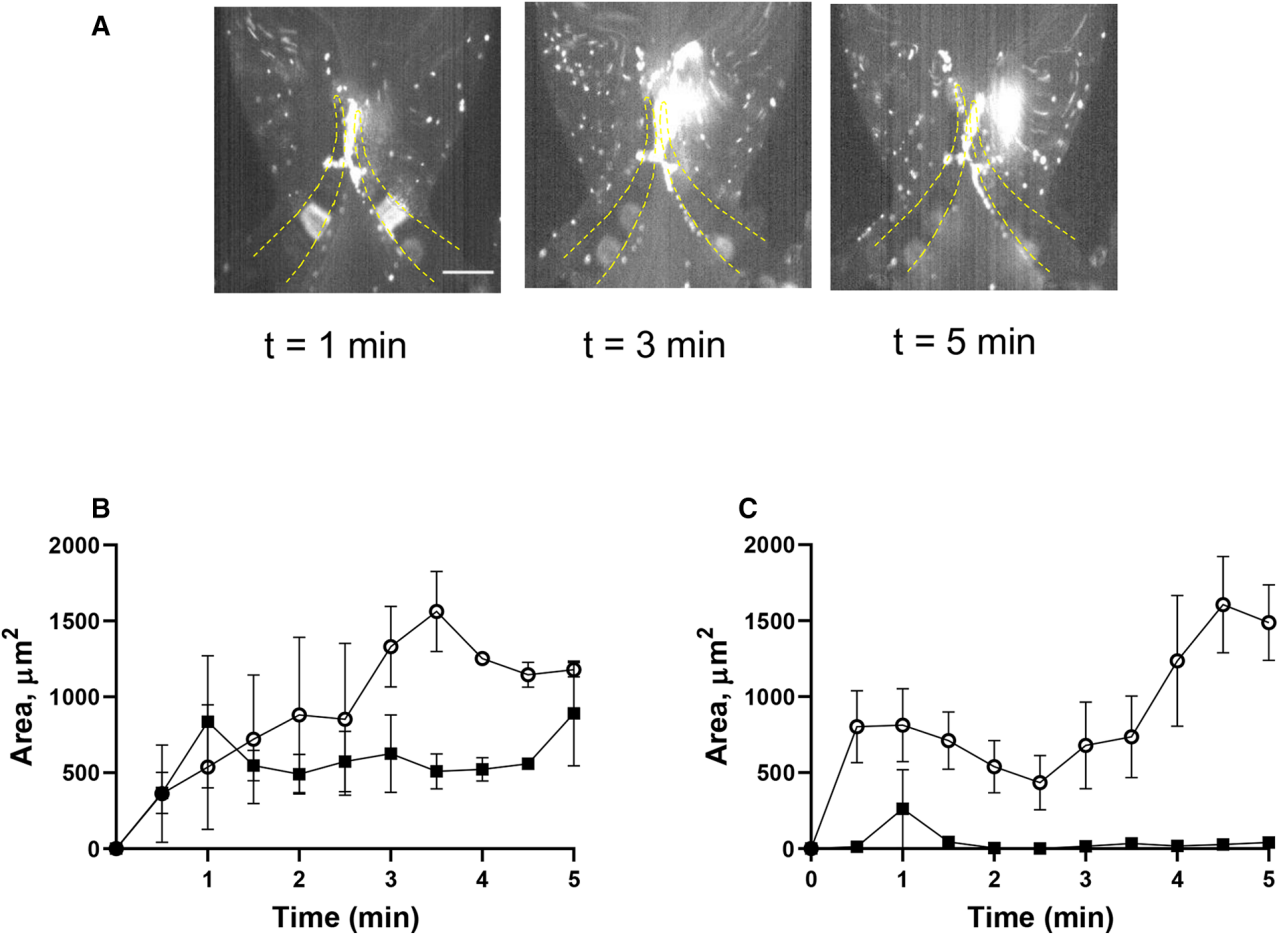
Histamine increases platelet deposition in the valve pockets (TS area). (**A**) HUVECs inside the chamber were incubated with histamine (10 µM, 15 min), and whole blood reconstituted with resting platelets was perfused through a symmetrical valve for 5 min. (**B,C**) quantitation of platelet accumulation on more flexible (circles) and less flexible (squares) leaflets, TL and TS region, respectively. Scale bar 100 µm. Error bars represent SEM, *n* = 3.

A key role of the endothelium in DVT initiation has been recently demonstrated in preclinical studies (10, 36). We therefore tested the effect of histamine, one of the most potent natural secretagogues of endothelial Weibel-Palade bodies, on platelet behavior in our system. Histamine treatment did not result in significant platelet accrual changes at the leaflets front side and their tips (TL area, AUC 4,126 ± 405 vs. 4,617 ± 1,119, *p* = 0.55). However, after histamine treatment, platelet aggregates developed predominantly at the valve pocket (TS area), where a thrombus is usually found in humans (AUC zero vs. 4,149 ± 505; Figures 5A–C; Supplementary Movie S8; AUC of TL and TS areas is presented in Supplementary Figure S1H). Thus, in sharp contrast to the system with unchallenged cells, platelet accumulation in the zone behind valve leaflets is likely not mediated by flow geometry only but requires either platelet or endothelial activation and Weibel-Palade body release as a critical component of its mechanism.

## 4. Discussion

In this study, we are presenting a model that combines mobile valve leaflets and pulsatile flow, characteristic of human veins, with the presence of an endothelial monolayer. This model recapitulates hemodynamics specific for venous flow and contains endothelium as a critical biological component involved in thrombosis prevention under physiological conditions and thrombosis initiation when prothrombotic stimuli start to prevail. The endothelial monolayer remains present and morphologically unchanged in human DVT (37), which implies that DVT is triggered by functional changes in the endothelial and blood cells rather than endothelial denudation, typical for thrombosis in arteries. Both the vessel wall and the blood cells also produce tissue factor (TF), an important component of the blood coagulation cascade involved in thrombosis. The investigation of the impact of TF on DVT using microfluidics would require further development of the model and should become a goal of future studies.

Hemodynamics in our model shares pivotal flow features in the presence of valves, such as two vortices described in another microfluidics model (28). The area around the second (“inner”) vortex has substantially lower oxygen level than the other vessel areas (28), which suggests that both endothelium and blood cells are exposed to hypoxia (7, 38). This results in endothelial activation, the release of Weibel-Palade bodies and recruitment of various cells including platelets, a process critical for thrombosis initiation (10, 11).

Platelet (C-type lectin-like receptor 2) CLEC-2 is crucial for experimental DVT (11), whereas platelet depletion prevents venous thrombosis development in mice (36). Also, procoagulant platelets, expressing phosphatidylserine, trigger blood clotting essential for thrombosis both in patients and *in vitro* (39). Thus, we have chosen platelet deposition as a readout representing the prothrombotic trend. Platelets were reconstituted in whole blood to create shear forces necessary for thrombus development (28). A pulsatile flow was utilized to mimic the flow pattern specific for veins and mainly generated due to the muscle pump, the dysfunction of which is associated with elevated risk for DVT (6). The involvement of this back-and-forth flow pattern, and especially its reflux component, in removal of forming fibrin and, as a result, in regulation of thrombus growth has recently been demonstrated (40).

Passage of reconstituted blood with resting platelets resulted in their accumulation predominantly at the tips and luminal part of the leaflets. Interestingly, shear stress, constantly changing both its direction and strength, did not destroy the aggregates suggesting their stability is likely based on receptor-ligand rather than simple electrostatic interactions. The kinetics of platelet deposition was remarkably similar to thrombus formation *in vivo* in the laser injury model, with rapid initial accumulation followed by gradual decrease (e.g., Figures 2E,F). Although the mechanism of this phenomenon remains incompletely understood, it is likely that insufficient fibrin formation cannot support stability of the growing clot. Of note, a strong correlation between stabilized platelet accumulation and maximal platelet accumulation was revealed, which implies that both platelet accretion and subsequent loss of some platelets are tightly regulated processes (41). Inhibition of *α*IIb*β*3 integrin with eptifibatide did not prevent deposition. This implies that the fibrinogen bridge between *α*IIb*β*3 integrins on two adjacent platelets, a mechanism involved in platelet aggregation by most agonists, is not implicated in platelet accumulation in our system. This is corroborated by the lack of platelet activation (including lack of activation of GPIIb-IIIa) following blood passage through the microfluidics chamber.

Activation of platelets with thrombin strongly increased platelet deposition on both leaflets of the symmetrical valve. Thrombin-activated platelets also accrued at the TS area of the leaflet, where human thrombi are usually found. Similar to resting platelets, deposition of activated cells not only was not reduced by inhibiting *α*IIb*β*3 integrin, but instead led to increased accumulation. This suggests that the exclusion of the *α*IIb*β*3-dependent mechanism likely increases the number of non-aggregated platelets available for adhesion (42). Increased platelet adhesion to a thrombogenic surface after blockade of the integrin has been reported (43). Inhibition of αIIb*β*3 by several antagonists including eptifibatide also increases the proportion of “coated” (collagen and thrombin-activated) platelets (44). Coated platelets are a fraction of the highly pro-thrombotic cells that form after simultaneous agonist activation of platelets with thrombin and collagen and express various procoagulant proteins, such as fibrinogen and von Willebrand factor on their surface (45). Our findings also demonstrate that hydrodynamics define the mechanism of platelet adhesion as an essential role of αIIb*β*3 in platelet deposition on HUVEC was reported under static conditions (46).

Lack of involvement of αIIb*β*3 in platelet recruitment in our model suggests the existence of another mechanism. Indeed, neutralization of GPIb*α* bending to the VWF A1 domain completely abolished the accumulation of activated platelets on both sides of the leaflets. This finding corroborates the previously published critical role of this interaction for DVT in mice (10). VWF is stored in both platelets and endothelial granules known as Weibel-Palade bodies. Activation of endothelial cells and Weibel-Palade body release are known prerequisites for venous thrombus development (10, 11). Local vein stimulation with histamine induces thrombosis even in mast cell-deficient mice, which are completely protected from DVT (12). In our model, histamine promoted platelet accumulation at the TS side of the leaflets, an effect likely mediated by endothelium-derived VWF and, potentially, other Weibel-Palade body constituents, such as P-selectin, whose role in experimental DVT has been reported (36). These data also suggest that results obtained in our *in vitro* model match reasonably well data from *in vivo* approaches. It is intriguing why histamine treatment did not affect platelet deposition at the TL area. The likely reason for this is dramatic difference in shear stress between TL and TS areas. VWF cleavage by ADAMTS13 requires its unraveling by shear forces (47, 48), which are much higher at the TL area. Also, blood exchange in the valvular pocket (TS area) is slower than between the leaflets (TL area), which leads to reduced supply of new portions of ADAMTS13 to the TS area. This results in accelerated cleavage of endothelium-derived VWF ultra-large VWF multimers at the TL area thereby reducing the effect of histamine on platelet accrual.

Interestingly, shear microgradient-induced formation of a thrombus consisting of only minimally activated discoid platelets has been reported (49). In this study, platelet accumulation was induced solely by flow dynamics and was likely independent of soluble platelet agonists, such ADP, thromboxane A2 and thrombin, and not associated with platelet shape change or degranulation, although we did not assess these parameters directly.

Blood coagulation plays a central role in venous thrombosis making anticoagulants a critical line of defense against DVT. Platelet trigger the coagulation cascade by expressing phosphatidylserine (PS), which provides a surface for clotting factor and thereby propagating thrombin generation and fibrin formation (50). Platelets also form a scaffold for fibrin fibers and modulate clot retraction (51, 52). Under low shear conditions, GPIb*α*—VWF interaction results in amplified PS expression, recruitment of coagulation factors, and accumulation of fibrin fibers at the platelet surface (53). Thus, enhanced platelet deposition observed in our experiments could trigger blood clotting leading to thrombus development, although the impact of this factor could be reduced since citrated (i.e., with low calcium level) blood was used. Although PS expression on resting platelets remained unchanged after passage through the flow system, the kinetics of fibrin formation and its dependency on platelets in our model is an interesting scientific question that should be addressed in future studies and, possibly, using alternative anticoagulants.

Venous valve flexibility is one of the essential characteristics affecting hemodynamics in the vein. Leaflet stiffness can increase because of diseases, such as phlebitis, in which increased deposition of connective tissue components, for example, collagen and elastin, makes the leaflet more rigid leading to its dysfunction and insufficiency (54). This could result in venous hypertension and reduced venous return to the heart directly affecting cardiac output (55). Moreover, stiffening of a valve may negatively affect hemodynamics at the neighboring healthy valve (56). We therefore explored platelet deposition on the valve leaflets with different ability to bend. Interestingly, preferable localization of adherent platelets depended on their activation status with resting platelets predominantly accruing at the luminal part of the more flexible leaflet, whereas accumulation of thrombin-activated platelets was moderately higher at the more rigid one. This finding suggests that increased rigidity of the leaflet renders it more prothrombotic when combined with hyperactivated platelets.

## 5. Conclusion

We have developed a microfluidics model recapitulating significant features of human veins and the Virchow's triad: specific flow pattern, flexible moving valves, and endothelial layer. Stimulation of either platelets or endothelium upregulates platelet deposition through a mechanism involving platelet-VWF interaction. This model could be useful in the venous thrombosis research field and help diminish the use of experimental animals in accordance with the 3R ethical principles.

## Data Availability

The original contributions presented in the study are included in the article/**Supplementary Material**, further inquiries can be directed to the corresponding authors.

## Supplementary material

Supplementary Video S1 Deposition of unchallenged platelets on symmetrical valve.

Supplementary Video S2 Deposition of unchallenged platelets on non-symmetrical valve.

Supplementary Video S3 Deposition of platelets treated by eptifibatide.

Supplementary Video S4 Deposition of platelets activated by thrombin.

Supplementary Video S5 Deposition of platelets activated by thrombin + eptifibatide.

Supplementary Video S6 Deposition of resting platelets treated with OS-1.

Supplementary Video S7 Deposition of thrombin-activated platelets treated by OS-1 peptide.

Supplementary Video S8 Deposition of platelets behind the leaflets is increased by histamine.

Supplementary Figure S1 AUC at TL and TS areas of flexible and less/non-flexible leaflets. AUC of platelets deposited at the TL and TS areas of (A) symmetrical valve, (B) non-symmetrical valve, (C) resting platelets + eptifibatide, (D) thrombin-activated platelets, (E) thrombin-activated platelets + eptifibatide, (F) resting platelets treated with OS-1, (G) thrombin-activated platelets treated with OS-1, and (H) histamine-treated HUVECs is presented as mean ± SD. Statistical comparison was performed by unpaired Student's t-test.

Supplementary Figure S2 Passage through the flow system does not activate platelets. Expression of CD41/GPIIb-IIIa, P-selectin, active CD41/GPIIb/IIIa (PAC-1), CD42b/GPIba and phosphatidylserine (annexin V) on platelets in the whole blood was analyzed before and after passage through the microfluidics chamber. Red dashed line represents the level of expression before the passage. Bars represent mean fluorescence intensity (MFI), mean ± SD. Statistical comparison was performed by unpaired Student's t-test.
