# Supplementary figures and images for: Platelet accumulation in an endothelium-coated elastic vein valve model of deep vein thrombosis is mediated by GPIbα—VWF interaction
Source: Front Cardiovasc Med. 2023 Apr 27;10:1167884. doi: 10.3389/fcvm.2023.1167884 (PMC10174463; doi:10.3389/fcvm.2023.1167884)

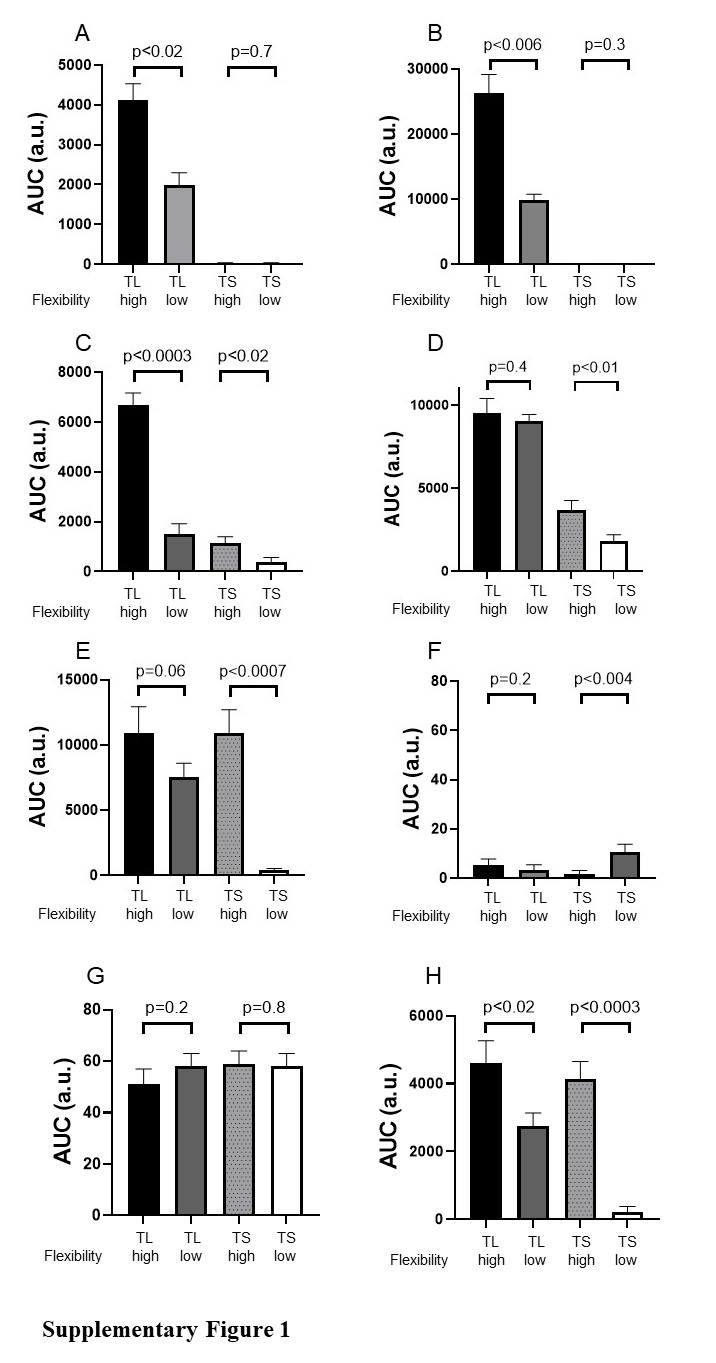

Supplement: Supplementary Figure S1 — AUC at TL and TS areas of flexible and less/non-flexible leaflets. AUC of platelets deposited at the TL and TS areas of (A) symmetrical valve, (B) non-symmetrical valve, (C) resting platelets + eptifibatide, (D) thrombin-activated platelets, (E) thrombin-activated platelets + eptifibatide, (F) resting platelets treated with OS-1, (G) thrombin-activated platelets treated with OS-1, and (H) histamine-treated HUVECs is presented as mean ± SD. Statistical comparison was performed by unpaired Student's t-test. [file Image1.jpg]

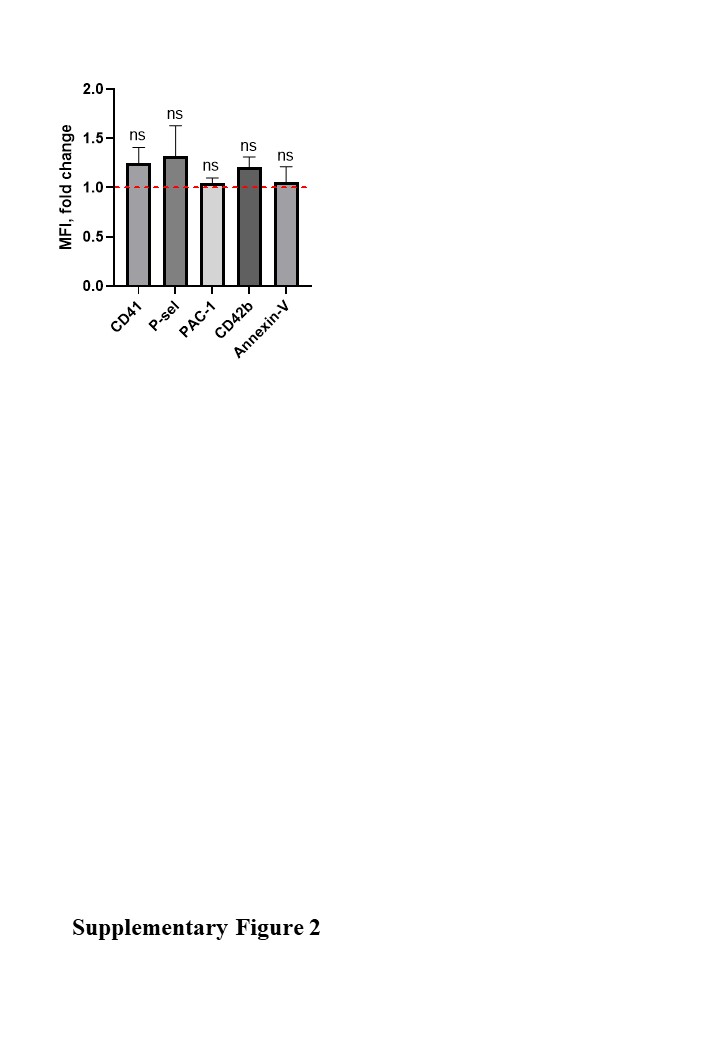

Supplement: Supplementary Figure S2 — Passage through the flow system does not activate platelets. Expression of CD41/GPIIb-IIIa, P-selectin, active CD41/GPIIb/IIIa (PAC-1), CD42b/GPIba and phosphatidylserine (annexin V) on platelets in the whole blood was analyzed before and after passage through the microfluidics chamber. Red dashed line represents the level of expression before the passage. Bars represent mean fluorescence intensity (MFI), mean ± SD. Statistical comparison was performed by unpaired Student's t-test. [file Image2.jpg]
